# Potential mechanism of action of Jing Fang Bai Du San in the treatment of COVID-19 using docking and network pharmacology

**DOI:** 10.7150/ijms.67116

**Published:** 2022-01-01

**Authors:** Jiaojiao Li, Kuo Zhang, Jimin Bao, Jingyu Yang, Chunfu Wu

**Affiliations:** 1 Department of Pharmacology, Shenyang Pharmaceutical University, 110016, Shenyang, PR China; 2 Department of Rehabilitation, Jin Qiu Hospital of Liaoning Province, 110016, Shenyang, PR China

**Keywords:** JingFangBaiDu San, COVID-19, network pharmacology, molecular docking

## Abstract

Coronavirus disease 2019 (COVID-19), caused by severe acute respiratory syndrome coronavirus 2 (SARS-CoV-2), severely infects people and has rapidly spread worldwide. JingFangBaiDu San (JFBDS) has been used to treat prevalent epidemic pathogens, common cold, headache, cough due to lung-cold, and other symptoms; however, its treatment for COVID-19 is unknown. Molecular docking and network pharmacology were applied to obtain ingredient-protein structures and the herb-ingredient-disease target network model, respectively, to explore the potential mechanism of JFBDS in COVID-19 treatment. Network pharmacology analysis showed that acacetin, wogonin, and isorhamnetin were the main active ingredients of JFBDS, and EGFR, PIK3CA, LCK, MAPK1, MAPK3, MAPK8, STAT3, TNF, IL2, and RELA were speculated to be crucial therapeutic targets. Moreover, the Toll-like receptors, HIF-1, PIK3K/AKT, MAPK, NF-κB and NOD-like receptor signaling pathways were important for JFBDS in COVID-19 treatment. Molecular docking analysis indicated that ingredients of JFBDS could bind to angiotensin converting enzyme II, spike protein, and chymotrypsin like protease (3CLpro), which inhibits virus entry and replication in host cells. This study provides a new perspective for understanding potential therapeutic effects and mechanisms of JFBDS in COVID-19 and may facilitate its clinical application.

## Introduction

People worldwide have been severely affected by the emergence of severe acute respiratory syndrome coronavirus 2 (SARS-CoV-2) since December 2019. Clinical manifestations of the coronavirus disease 2019 (COVID-19) are fever, cough, shortness of breath, muscle ache, confusion, headache, sore throat, rhinorrhea, chest pain, diarrhea, nausea, and vomiting. Most infected patients developed acute respiratory distress syndrome and eventually died of multiple organ failure 1, 2. SARS-CoV-2 is deadlier than Middle East respiratory syndrome coronavirus and SARS-CoV. Unlike SARS-CoV, SARS-CoV-2 can generate 3.2 fold more infectious virus particles from infected lung tissues, which indicates its higher infectivity in human lung tissues 3. Various vaccines, such as virus vectored, protein subunit, genetic, and monoclonal antibody hindrances vaccines have been developed to date 4. Although vaccines have recently been used in clinical practices, they need more time to gain popularity.

Traditional Chinese medicine (TCM) can protect COVID-19 patients from tissue injury, which is attributed to its anti-inflammatory, antioxidant, and anti-apoptotic effects 5. TCM has been used to treat acute respiratory tract infections, acute bronchitis, influenza, measles, sore throat, common cold and SARS 6. According to the Diagnosis and Treatment Protocol for Novel Coronavirus Pneumonia (Trial Version 7) in China 7, several Chinese herbal formulas, including Qing Fei Pai Du Decoction (QFPD), Lian Hua Qing Wen capsule (LHQW), Shen fu decoction (SF), and Xuebijing injection (XBJ) have been shown to be beneficial for COVID-19 treatment. Among multiple prescriptions for epidemic pathogens in ancient China, JingFangBaiDu San (JFBDS), an old prescription documented in “*She Sheng Zhong Miao Fang”* written by Shiche Zhang during the Ming dynasty deserves attention. JFBDS is used for exogenous febrile disease, prevalent epidemic pathogens, common cold, headache, dizziness, restlessness, body pain, cough due to lung-cold and other symptoms in clinical practice. The indications for JFBDS include cold dampness pathogenic factors, epidemic diseases, dysentery, sores, common cold and influenza in clinical practice 8, which are consistent with symptoms of COVID-19.

Network pharmacology has been widely applied in TCM studies in recent years 9-13. This could transform TCM from an experience-based to an evidence-based medicine system and hasten drug discovery 14. The Traditional Chinese Medicine Systems Pharmacology Database and Analysis Platform (TCMSP) is widely applied as a unique pharmacology platform for Chinese herbal medicines that captures relationships between drugs, targets, and diseases from TCM researches done using network pharmacology. Many studies have indicated that angiotensin-converting enzyme II (ACE2), spike protein (S1), and chymotrypsin like protease (3CLpro) play an important role in virus infection and replication. However, mechanisms of action of JFBDS against COVID-19 are unknown and ingredients interactions with COVID-19 targets have not been reported.

In this study, we constructed an herb-ingredient-disease target network model using network pharmacology to elucidate the possible effect of JFBDS in the treatment of COVID-19. Ingredients of JFBDS and target of COVID-19 were entered into Cytoscape to present herb-ingredient-disease target network model. Moreover, the intersectant targets (named ingredient-disease-targets) made from intersecting ingredient-associated targets and COVID-19-associated disease targets were analyzed using DAVID database. Finally, to verify the validity of the predicted results, molecular docking was used to analyze the interaction of ingredients with ACE2, S1, and 3CLpro.

## Methods and Materials

### Experimental design

**Figure 1:**
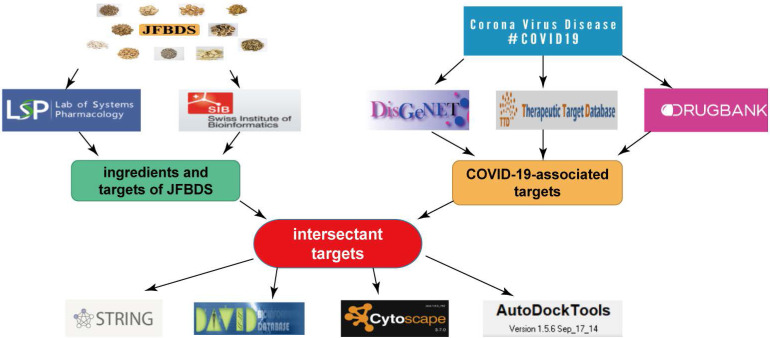
The experiment design of JFBDS on COVID-19 by network pharmacology and molecular docking

The experimental design is shown in Figure 1. Ingredients of JFBDS were acquired using TCMSP and targets of these ingredients were predicted by Swiss Target Prediction (http://swisstargetprediction.ch/). COVID-19 associated disease targets were mined using DisGeNet (https://www.disgenet.org), TTD (http://db.idrblab.net/ttd), and Drug Bank (https://www.drugbank.ca). Subsequently, ingredient-disease-targets were analyzed using DAVID database. These data were entered into Cytoscape to present the herb-ingredient-disease target network model.

### Collection of ingredients in JFBDS

JFBDS containes eleven herbs, including *Bupleurum chinense* DC. (Chaihu), *Ligusticum chuanxiong* Hort*.* (Chuanxiong), *Angelica pubescens* Maxim. f.* biserrata* Shan et Yuan (Duhuo), *Saposhnikovia divaricata* (Turcz.) Schischk. (Fangfeng), *Poria cocos* (Schw.) Wolf (Fuling), *Glycyrrhiza uralensis* Fisch. (Gancao), *Schizonepeta tenuifolia* Briq*.* (Jingjie), *Platycodon grandiflorum* (Jacq.) A. DC. (Jiegeng), *Peucedanum praeruptorum* Dunn (Qianhu), *Notopterygium inchum* Ting ex H.T. Chang (Qianghuo) and *Citrus aurantium* L*.* (Zhiqiao). The quantity of Gancao in this prescription was 1.5 g and that of other herbs was 4.5 g. The instructions in TCMSP, conditions of oral bioavailability (OB) ≥ 30% and drug-likeness (DL) ≥ 0.18 were limitations to obtaining the potential ingredients.

### Targets prediction of ingredients in JFBDS

Targets of ingredients in JFBDS were obtained by entering the SMILES numbers of each ingredient into the Swiss Target Prediction basement, which predicted targets of the ingredients based on 2D and 3D structures of the ingredient.

### Collection of COVID-19-associated targets

DisGeNet, TTD and Drug Bank were used to collect COVID-19-associated targets by searching novel coronavirus, Pneumonia and cytokine-related in above three databases.

### Construction of network and bioinformatics analysis

Intersectant targets were obtained by intersecting the ingredient-associated targets and COVID-19-associated disease targets. The ingredient-disease-targets were entered into STRING to obtain the protein-protein interaction (PPI) network. To display the relationship between herb, ingredient, and ingredient-disease-target, the herb-ingredient-disease target network model was created using Cytoscape. Gene Ontology (GO) annotation and Kyoto Encyclopedia of Genes and Genomes (KEGG) pathway analyses were performed using DAVID (https://david.ncifcrf.gov/). The results are presented as bubble charts drawn using OmicShare platform (https://www.omicshare.com/tools/).

### Ingredient-protein molecular docking

3D structures of the ACE2 (PDB ID: 6M2N), S1 (PDB ID:7BZ5) and 3CLpro (PDB ID: 6LU7) were downloaded from the RCSB PDB database (https://www.rcsb.org/). AutoDock Tools 1.5.6 software was used to process protein structures, including removing water molecules, adding hydrogen, calculating Gasteiger charges. The PubChem database (https://pubchem.ncbi.nlm.nih.gov/) was used to download the two-dimensional (2D) structures of the top ten compounds. The 2D structure was processed and converted into the PDB format using Chem3D. Structures of the three proteins (ACE2, S1, and 3CLpro) and their ligands were obtained using PyMol 2.4. Ten ingredients and three proteins were produced using molecular docking. Finally, the three proteins were produced with their original ligands using molecular docking. Binding energies were obtained and visualized using PyMol 2.4.

## Results

### Collection of chemical ingredients from JFBDS and predicting of potential targets

Chemical ingredients of these herbs were collected using TCMSP, which contained 220 active ingredients. The representative active ingredients are shown in Table 1, and detailed chemical ingredient information is provided in the supplementary materials. The structural information of 25 chemical ingredients was not available; therefore, their targets could not be predicted. Potential targets of the remaining 195 chemical ingredients were predicted using by Swiss Target Prediction, and 1073 potential targets were collected. The representative potential targets are shown in Table 2, and detailed target information is provided in the supplementary materials.

### Collection of COVID-19-associated targets

DisGeNet, TTD, and Drug Bank were used to search for COVID-19 targets. Keywords including COVID-19, pneumonia, and cytokine-related syndrome were used to obtain 747 targets. The information on partial COVID-19 associated targets is shown in Table 3, while detailed target information is provided in the supplementary materials.

### Collection of ingredient-disease-targets and analysis of PPI network

**Figure 2:**
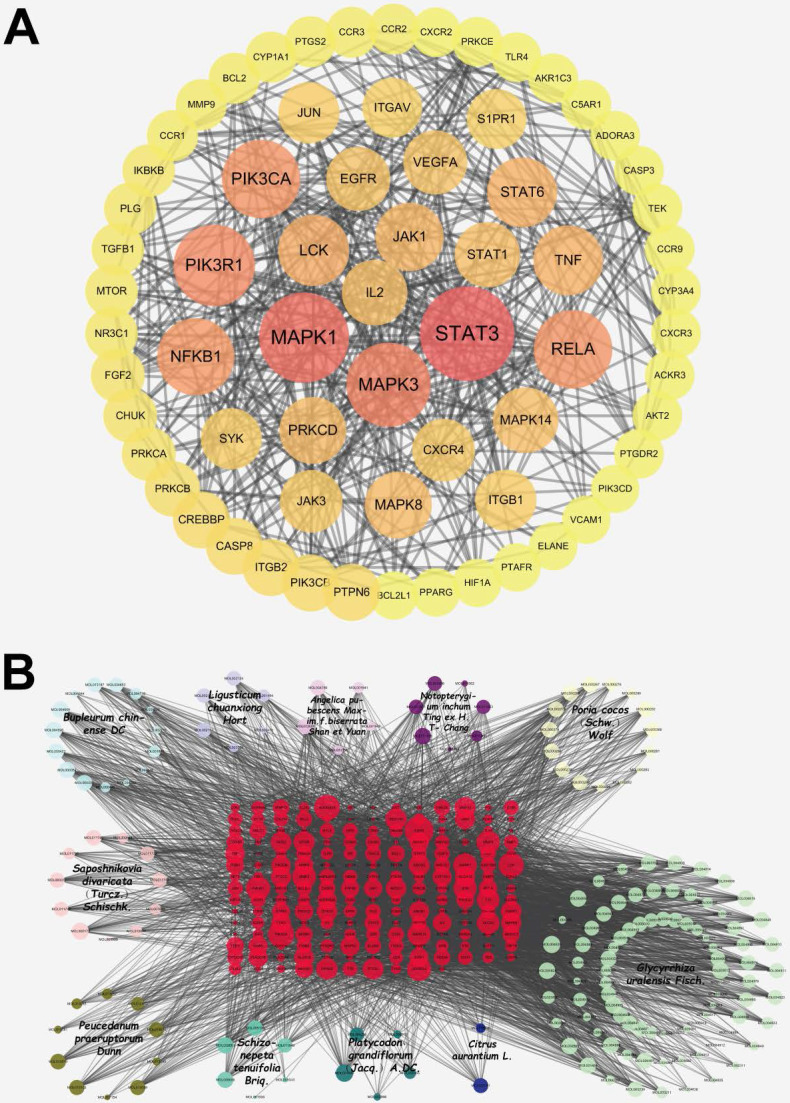
The PPI network of ingredient-disease-targets (A). The interaction network of herb-ingredient-disease targets (B). Among this, red color nodes represented ingredient-disease-targets; light blue nodes represented *Bupleurum chinense* DC. (Chaihu), dark bule nodes represented *Citrus aurantium* L*.* (Zhiqiao), light purple nodes represented *Ligusticum chuanxiong* Hort*.* (Chuanxiong), dark purple nodes represented *Notopterygium inchum* Ting ex H.T. Chang (Qianghuo), light pink nodes represented *Saposhnikovia divaricata* (Turcz.) Schischk. (Fangfeng), dark pink nodes represented *Angelica pubescens* Maxim.f*.biserrata* Shan et Yuan (Duhuo), light yellow nodes represented *Poria cocos* (Schw.) Wolf (Fuling), dark yellow nodes represented *Peucedanum praeruptorum* Dunn (Qianhu), light green nodes represented *Glycyrrhiza uralensis* Fisch. (Gancao), dark green nodes represented *Platycodon grandiflorum* (Jacq.) A. DC. (Jiegeng) and bright green nodes represented *Schizonepeta tenuifolia* Briq*.* (Jingjie).

According to the method, 192 ingredient-disease-targets were obtained. Thereafter, the STRING database was used to obtain a PPI network of 192 interactive targets by limiting the highest degree (degree ≥ 0.9) of confidence level and eliminating independent target proteins. This network finally contained 192 nodes and 772 edges (Figure 2A), in which nodes represent the target, edges represent the relationship between the targets, and different colors represent different cluster interactions. To display the PPI network intuitively, the degree values from 0 to 9 were hidden (Figure 2A). The top ten targets of the degree values are displayed in the supplementary materials.

### Creation of herb-ingredient-disease targets network in JFBDS

As shown in Figure 2B, the network of herb-ingredient-disease targets was built using Cytoscape. 360 nodes and 3901 edges were obtained in the herb-ingredient-disease targets network. According to the degree in network, the top ten ingredients were MOL001689 (acacetin), MOL004835 (glypallichalcone), MOL000173 (wogonin), MOL004905 (glyuranolide), MOL004598 (3',4',5',3,5,6,7-Heptamethoxy flavone), MOL004828 (glepidotin A), MOL004856 (gancaonin A), MOL005013 (18α-hydroxy glycyrrhetic acid), MOL000354 (Isorhamnetin) and MOL002565 (medicarpin). The top targets were mainly EGFR, PIK3CA, LCK, MAPK1, MAPK3, MAPK8, STAT3, TNF, IL2, and RELA. This suggests that JFBDS has therapeutic effects on COVID-19 probably through the interaction between the ingredients and targets. Detailed information on the top ten ingredients and targets are shown in Tables 4 and 5.

### Bioinformatics of ingredient-disease-target

**Figure 3:**
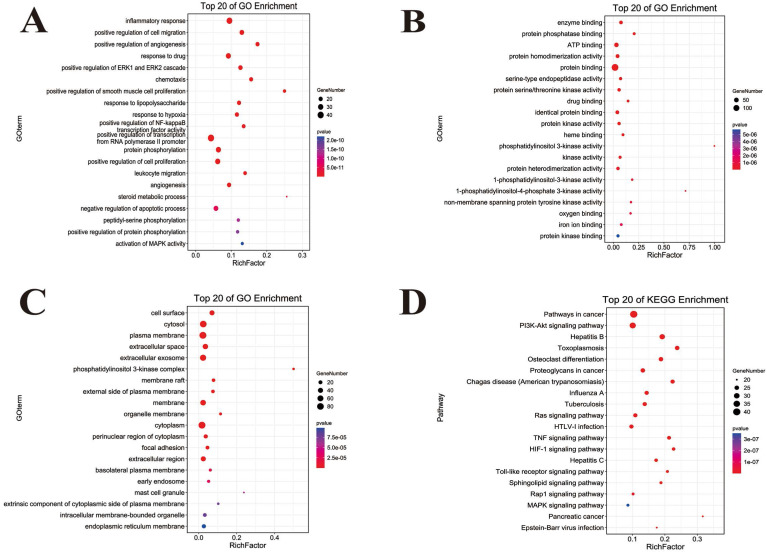
GO and KEGG functional annotation analysis of ingredient-disease-targets in JFBDS for treating COVID-19. GO enrichment of putative targets was divided into biological process (A), molecular function (B) and cellular component (C). The pathway of putative targets was predicted by KEGG enrichment (D).

**Figure 4:**
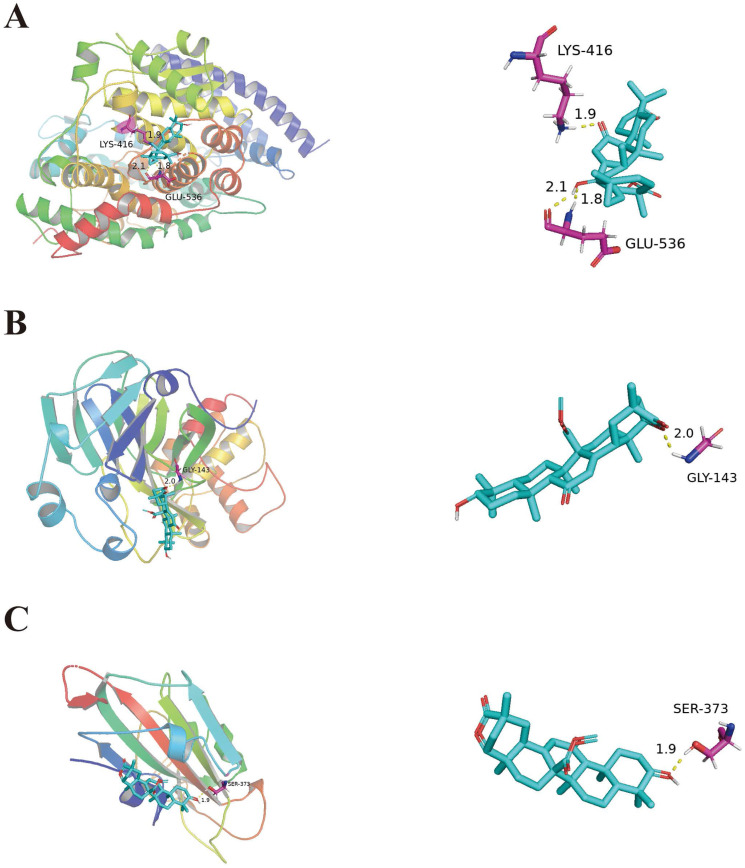
Molecular docking of 18α-hydroxy glycyrrhetic acid bound with ACE2 (A). Molecular docking of Glyuranolide bound with 3CLpro and S1(B, C).

The GO function enrichment analysis of ingredient-disease-targets was performed using DAVID in the categories of biological process (BP), molecular function (MF) and cellular component (CC) are shown in Figure 3. In BP term, the results showed that inflammatory response, chemotaxis, response to lipopolysaccharide (LPS) positive regulation of NF-κB transcription factor activity, and leukocyte migration played important roles in the treatment of COVID-19. In MF terms, the results demonstrated that ATP and protein binding were two important molecular functions. In CC terms, the cytosol, plasma membrane and cytoplasm were the main sites of the targets. To uncover the potential therapeutic mechanisms of JFBDS in COVID-19, the ingredient-disease-targets were analyzed using KEGG. The top 20 pathways of KEGG enrichment analysis are shown in Figure 4D. Among these, the most significant were the Toll-like receptors, HIF-1, PIK3K/AKT, MAPK, NF-κB and NOD-like receptor signaling pathways.

### Results of molecular docking

Molecular docking was used to verify the impact of top ten ingredients on ACE2, S1, and 3CLpro. The binding energies of ACE2, S1, and 3CLpro with their original ligands were -2.51 kJ/mol, -4.6 kJ/mol and -5.44 kJ/mol, respectively. These results indicate that the top ten ingredients have a strong affinity for ACE2, S1, and 3CLpro. For instance, 18α-hydroxy glycyrrhetic acid was strongly bound to ACE2. Glyuranolide was strongly associated with S1 and 3CLpro. As shown in Figure 4, 18α-hydroxy glycyrrhetic acid was bound to ACE2 through three hydrogen bonds with the amino acid residues Lys416 and Glu536. Glyuranolide was bound to S1 and 3CLpro by one hydrogen bond with the amino acid residues Gly143 and Ser373, respectively. The binding energies of various compounds are shown in Table 6.

## Discussion

COVID-19 is a clinical syndrome caused by SARS-CoV-2 that has been sweeping the globe since December 2019. COVID-19 may result in cytokine storm syndrome and acute respiratory distress syndrome 15. Cytokine storms contain several disorders of immune dysregulation characterized by systemic inflammation and multiple organ dysfunction 16, which results in the clinical deterioration of COVID-19 patients. Many cytokines played a crucial role in COVID-19, such as IL-2, IL-6, IL-7, IL-8, IL-1β, IL10 and TNF-α 2, 17. Moreover, many signaling pathways, including the toll-like receptors, NF-κB, MAPK, and TNF, are involved in COVID-19 18, 19. Overcoming this epidemic has become the toughest issue worldwide. Currently, antiviral therapy is widely used in clinical practices, including alpha-interferon, lopinavir/ritonavir, ribavirin, chloroquine phosphate, and arbidol 20. However, these medicines have some side effects, and the combination of these medicines is not recommended 21. Despite the partial use of vaccines, effective drug development remains an urgent task.

The TCM treatment approach has been widely used to treat COVID-19 in China. Many studies have reported that TCM could resist viral pneumonia by modulating cytokine production, controlling viral replication, and regulating immunological function 22. According to the COVID-19 seventh edition diagnostic criteria, four prescriptions, QFPD, LHQW, SF, and XBJ, achieved satisfactory results 13, 19, 23, 24. Among them, QFPD was reported to exhibit immune regulation, anti-infection, anti-inflammation and multi-organ protection by regulating toll-like receptors, NF-κB, and MAPK signaling pathways, which subsequently inhibits the release of pro-inflammatory factors 18, 23. SF and XBJ inhibit inflammation and regulate immunity. XBJ also alleviated pneumonia-induced multi-organ damage 19, 24. LHQW significantly inhibited SARS-CoV-2 replication and diminished the cytokine release 13. These data indicate that TCM plays an irreplaceable role in immune regulation, anti-infection, anti-inflammation, and multi-organ protection. JFBDS contains eleven herbs that have been used to treat cough, influenza, and respiratory tract infections for nearly four hundred years. According to the indications of JFBDS, it could treat epidemic pathogens and lung diseases, improve digestive tract symptoms, and relieve pain, which is consistent with most symptoms of COVID-19.

According to the degree value of the targets, several potential active ingredients that played an important role in the treatment of COVID-19, including acacetin, glypallichalcone, wogonin, gancaonin A, and isorhamnetin were screened in JFBDS. Acacetin was obtained from *Schizonepeta tenuifolia* Briq. Glypallichalcone and Gancaonin A were obtained from *Glycyrrhiza uralensis* Fisch. Wogonin was obtained from* Saposhnikovia divaricata* (Turcz.) Schischk. Isorhamnetin was obtained from both *Glycyrrhiza uralensis* Fisch. and *Bupleurum chinense* DC. Acacetin is a flavone that has anti-inflammatory effects 25, 26. It has been reported to decrease the activity of cyclooxygenase-2 (COX-2) and nitric oxide synthase (NOS) 27, suppress levels of proinflammatory cytokines and chemokines, reduced the adhesion of eosinophils to tracheal epithelial cells 28, and inhibit sepsis-induced acute lung injury 26, 29. Wogonin is also a flavonoid with anti-inflammatory, anti-fibrotic, and anti-cancer effects 30. Some studies have shown that the possible mechanism of action of wogonin on inflammation was associated with the reduction of phosphorylation and activation of transcription factors STAT1 or STAT3 31. Moreover, wogonin inhibits the expression of COX-2 and HIF-1α and reverses hypoxia resistance by regulating the PI3K/Akt signaling pathway 21, 32. As one of the major metabolites of quercetin, isorhamnetin has anti-inflammatory activity and reduces the expression of inflammatory genes in macrophages stimulated by LPS 33. It also significantly inhibits the inflammation, proliferation and migration of BEAS-2B cells by regulating the MAPK and NF-κB signaling pathways 34. Therefore, it is reasonable to speculate that acacetin, wogonin, and isorhamnetin are the main ingredients of JFBDS that treat COVID-19 by regulating PI3K/AKT, STAT3, MAPK, and NF-κB signaling pathways.

According to the results of cytoscape, the targets of JFBDS for treating COVID-19 mainly contained EGFR, PIK3CA, lymphocyte-specific protein tyrosine kinase (LCK), mitogen-activated protein kinase 1 (MAPK1), MAPK3, MAPK8, STAT3, TNF, IL2 and RELA. These targets were mainly involved in toll-like receptors, HIF-1, PIK3K/AKT, NF-κB and NOD-like receptor signaling pathways, which have important effects on cytokines. For example, EGFR is related to pulmonary inflammation and its phosphorylation can regulate the expression of pro-inflammatory factors, such as IL-8 and CXCL1 35, 36. EGFR also activates multiple signaling pathways, such as PI3K/Akt/mTOR, JAK/STAT and NF-κB, which are closely related to inflammation 37. LCK, a cytoplasmic tyrosine kinase expressed in T cells and natural killer cells, is a necessary step to activate T cell. Therefore, selective inhibition of LCK can produce immunosuppressive effects 38. As an important pathway for extracellular signal transduction into the nucleus, MAPK cascade plays an important role in cell proliferation, differentiation and apoptosis 39. MAPK1, MAPK3, MAPK8 and MAPK14 can be activated by stimulating phosphorylation during changes in the internal environment, such as osmotic pressure changes, oxidative stress, inflammatory factors and viral infection. By suppressing MAPK signalling pathways, flavonoids inhibit the production of inflammatory cytokines in LPS-stimulated macrophages 40. IL-2, with multiple effects on the immune system, was considered a key cytokine because of its regulatory effects on T cells. IL-2 performs crucial functions during immune homeostasis, which can optimize and regulate lymphocyte responses 41. IL-2 can also activate the STAT, PI3K/AKT and MAPK signaling pathways 42. In GO terms analysis, it was found that inflammatory response, chemotaxis, response to LPS, positive regulation of NF-κB transcription factor activity and leukocyte migration might play an important role in the treatment of COVID-19. KEGG pathway analysis revealed that the toll-like receptors, HIF-1, PIK3K/AKT, MAPK, NF-κB, and NOD-like receptor signaling pathways had significant effects on COVID-19 therapy. The toll-like receptors and PI3K/Akt signaling pathways have already been identified as important for anti-inflammatory effects on COVID-19 18, 24. The activation of PI3K/Akt/mTOR pathway causes pulmonary fibrosis and lung injury by increasing lung fibroblasts and lung epithelial cells 13. This indicates that it might be closely related to the development and treatment of COVID-19 43.

**Figure 5:**
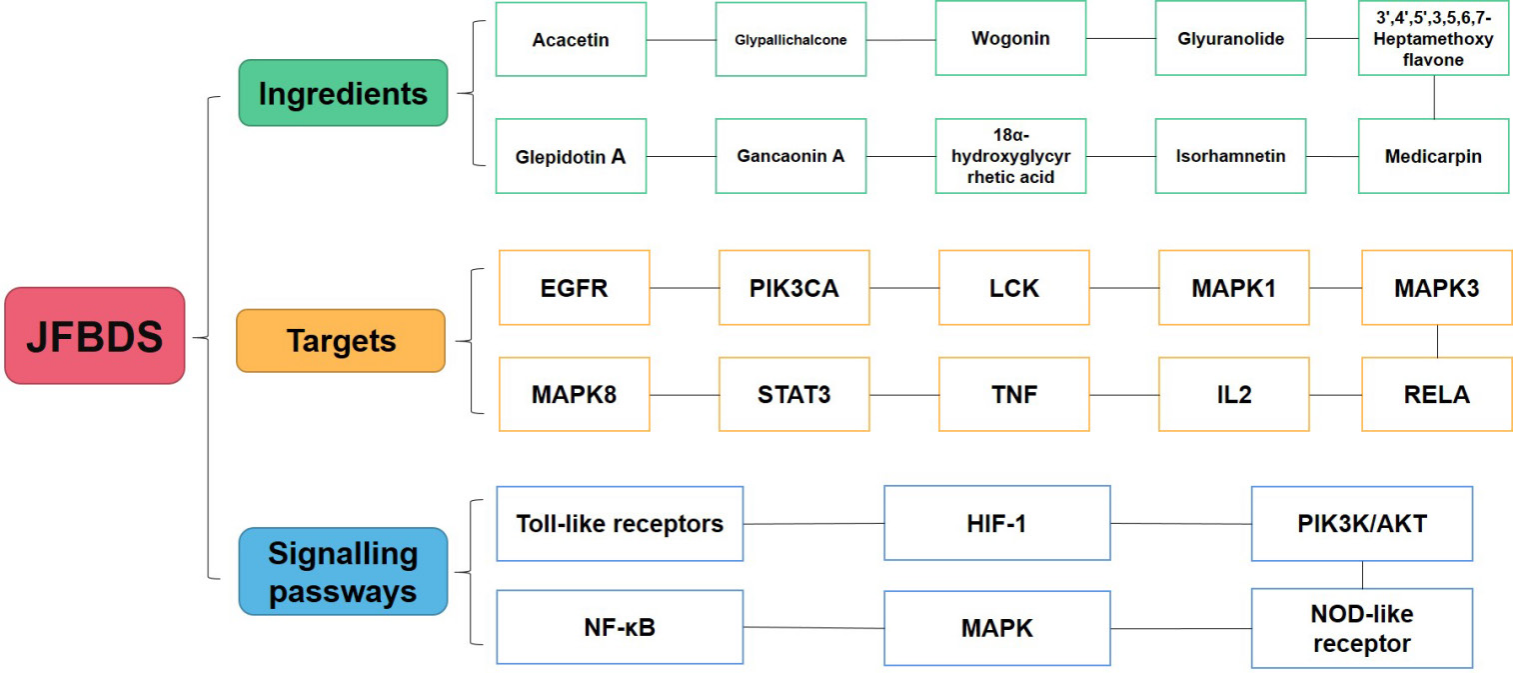
The ingredients, targets and signalling pathway of JFBDS on COVID-19.

Some studies have reported targets closely related to COVID-19. ACE2 mainly exists among he epithelial cells of alveoli, trachea, bronchus, serous bronchial glands, alveolar monocytes, and macrophages of the lower respiratory tract. It is the cellular receptor of COVID-19 44. The entry of SARS-CoV-2 into cells requires S1 binding to the cell surface receptor. S1 also facilitates the attachment of target cells to the viral surface of the S protein 45. 3CLpro is present in the multi-protein ORF1Ab of SARS-CoV-2 and is an attractive target against SARS-CoV-2 because of its importance in the replication of the virus^46^. Therefore, the main ingredients of high degree were docked with ACE2, 3CLpro, and S1 proteins. In the molecular docking process, lower the binding energy, more effective is the molecular association. The ingredient-protein interaction with docking energy less than 5 kJ/mol signified effective docking 47. According to the results of molecular docking, the sectional ingredients showed a strong binding affinity to the three proteins. In particular, 18α-hydroxy glycyrrhetic acid and glyuranolide showed high affinity, indicating that both were important active ingredients of JFBDS for COVID-19. Glypallichalcone, glyuranolide, glepidotin A, gancaonin A, 18α-hydroxy glycyrrhetic acid, medicarpin, and isorhamnetin were seven of the top ten ingredients with high binding energy that were derived from *Glycyrrhiza uralensis* Fisch. However, isorhamnetin was also derived from *Bupleurum chinense* DC. Therefore, *Glycyrrhiza uralensis* Fisch. took an irreplaceable position in JFBDS 48. The ingredients, targets, and signaling pathways of JFBDS in COVID-19 are shown in Figure 5. However, exact ingredients and therapeutic targets of JFBDS need to be studied further and confirmed.

## Conclusion

In this study, 220 active ingredients of JFBDS were collected using TCMSP and 192 ingredient-disease-targets were collected from Swiss Target Prediction database, DisGeNet, TTD, and DrugBank. Bioinformatics analysis revealed that acacetin, wogonin, and isorhamnetin were main active ingredients of JFBDS for the treatment of COVID-19. EGFR, PIK3CA, LCK, MAPK1, MAPK3, MAPK8, STAT3, TNF, IL2, and RELA were crucial therapeutic targets for the treatment of COVID-19 using JFBDS. Moreover, the toll-like receptors, HIF-1, PIK3K/AKT, MAPK, NF-κB, and NOD-like receptor signaling pathways were involved in the treatment of COVID-19. The partially predicted ingredients of JFBDS were verified using molecular docking. This study provides a new perspective for understanding the potential therapeutic effect and mechanism of action of JFBDS on COVID-19 and facilitates its clinical application.

## Supplementary Material

Supplementary information.

## Figures and Tables

**Table 1:** The information of partial active ingredients in JFBDS

Herb	MOL ID	Ingredient	OB%	DL
*Bupleurum chinense* DC. (Chaihu)	MOL001645	Linoleyl acetate	42.1	0.2
	MOL000449	Stigmasterol	43.83	0.76
	MOL000354	isorhamnetin	49.6	0.31
	MOL001645	Linoleyl acetate	42.1	0.2
*Ligusticum chuanxiong* Hort*.* (Chuanxiong)	MOL001494	Mandenol	42	0.19
	MOL002135	Myricanone	40.6	0.51
	MOL002140	Perlolyrine	65.95	0.27
	MOL002151	senkyunone	47.66	0.24
*Angelica pubescens* Maxim.f.*biserrata* Shan et Yuan (Duhuo)	MOL001941	Ammidin	34.55	0.22
	MOL001942	isoimperatorin	45.46	0.23
	MOL000358	beta-sitosterol	36.91	0.75
	MOL003608	O-Acetylcolumbianetin	60.04	0.26
*Saposhnikovia divaricata* (Turcz.) Schischk. (Fangfeng)	MOL000011	(2R,3R)-3-(4-hydroxy-3-methoxy-phenyl)-5-methoxy-2-methylol-2,3-dihydropyrano[5,6-h] 1,4 benzodioxin-9-one	68.83	0.66
	MOL011730	11-hydroxy-sec-o-beta-d-glucosylhamaudol_qt	50.24	0.27
	MOL011732	anomalin	59.65	0.66
	MOL011737	divaricatacid	87	0.32
*Poria cocos* (Schw.) Wolf (Fuling)	MOL000273	(2R)-2-[(3S,5R,10S,13R,14R,16R,17R)-3,16-dihydroxy-4,4,10,13,14-pentamethyl-2,3,5,6,12,15,16,17-octahydro-1H-cyclopenta[a]phenanthren-17-yl]-6-methylhept-5-enoic acid	30.93	0.81
	MOL000275	trametenolic acid	38.71	0.8
	MOL000276	7,9(11)-dehydropachymic acid	35.11	0.81
	MOL000279	Cerevisterol	37.96	0.77
*Glycyrrhiza uralensis* Fisch. (Gancao)	MOL001484	Inermine	75.18	0.54
	MOL001792	DFV	32.76	0.18
	MOL000211	Mairin	55.38	0.78
	MOL002311	Glycyrol	90.78	0.67
*Schizonepeta tenuifolia* Briq*.* (Jingjie)	MOL011849	Schizonepetoside B	31.02	0.28
	MOL011856	Schkuhrin I	54.45	0.52
	MOL002881	Diosmetin	31.14	0.27
	MOL000359	sitosterol	36.91	0.75
*Platycodon grandiflorum* (Jacq.) A. DC. (Jiegeng)	MOL001689	acacetin	34.97	0.24
	MOL004355	Spinasterol	42.98	0.76
	MOL004580	cis-Dihydroquercetin	66.44	0.27
	MOL005996	2-O-methyl-3-O-β-D-glucopyranosyl platycogenate A	45.15	0.25
*Peucedanum praeruptorum* Dunn (Qianhu)	MOL013076	(8S,9R)-9-hydroxy-8-(2-hydroxypropan-2-yl)-8,9-dihydrofuro[2,3-h] chromen-2-one	37.3	0.2
	MOL013077	Decursin	39.27	0.38
	MOL013078	praeruptorin E	51.22	0.66
	MOL013079	dl-praeruptorin a	46.46	0.53
*Notopterygium inchum* Ting ex H.T. Chang (Qianghuo)	MOL001941	Ammidin	34.55	0.22
	MOL011962	6'-Feruloylnodakenin	32.02	0.67
	MOL011963	8-geranoxy-5-methoxypsoralen	40.97	0.5
	MOL011968	coumarin,glycoside	33.07	0.78
*Citrus aurantium* L*.* (Zhiqiao)	MOL013381	Marmin	38.23	0.31
	MOL002341	Hesperetin	70.31	0.27
	MOL000358	beta-sitosterol	36.91	0.75
	MOL004328	naringenin	59.29	0.21

**Table 2:** The information of partial potential targets in JFBDS

Gene Symbol	UniProt ID	Gene Symbol	UniProt ID
ALOX12	P18054	MMP3	P08254
BTK	Q06187	MMP9	P14780
CD81	P60033	NFKB1	P19838
CTSK	P43235	NLRP3	Q96P20
NOD2	Q9HC29	CASP1	P29466
RELA	Q04206	CASP8	Q14790
ICAM1	P05362	MAPK3	P27361
IKBKB	O14920	JUN	P05412
IL2	P60568	PIK3CA	P42336
IRAK4	Q9NWZ3	PIK3R1	P27986
TLR4	O00206	STAT1	P42224
TLR9	Q9NR96	STAT3	P40763
TNF	P01375	TBK1	Q9UHD2
VCAM1	P19320	SYK	P43405
MAPK1	P28482	JAK1	P23458

**Table 3:** The information of partial COVID-19-associated targets

Gene Symbol	UniProt ID	Gene Symbol	UniProt ID
CD19	P15391	TNF	P01375
CRP	P02741	IL13	P35225
IFNG	P01579	CSF2	P04141
IL6	P05231	IL4	P05112
TWIST1	Q15672	CXCR2	P25025
CARTPT	Q16568	IL33	O95760
IL1B	P01584	CXCL5	P42830
CXCL1	P09341	HMOX1	P09601
CCL2	P13500	MYD88	Q99836
CCL11	P51671	TGFB1	P01137
IL18	Q14116	CCL24	O00175
CCL17	Q92583	CASP1	P29466
ITGB2	P05107	SIRT1	Q96EB6
TLR4	O00206	IL6ST	P40189
IL17A	Q16552	CD40LG	P29965

**Table 4:** The information of top ten ingredients of degree values from analysis cytoscape

MOL ID	Molecule Name	Herb
MOL001689	Acacetin	*Platycodon grandiflorum* (Jacq.) A. DC. (Jiegeng)
MOL004835	Glypallichalcone	*Glycyrrhiza uralensis* Fisch. (Gancao)
MOL000173	Wogonin	*Saposhnikovia divaricata* (Turcz.) Schischk. (Fangfeng)
MOL004905	Glyuranolide	*Glycyrrhiza uralensis* Fisch. (Gancao)
MOL004598	3',4',5',3,5,6,7-Heptamethoxy flavone	*Bupleurum chinense* DC. (Chaihu)
MOL004828	Glepidotin A	*Glycyrrhiza uralensis* Fisch. (Gancao)
MOL004856	Gancaonin A	*Glycyrrhiza uralensis* Fisch. (Gancao)
MOL005013	18α-hydroxy glycyrrhetic acid	*Glycyrrhiza uralensis* Fisch. (Gancao)
MOL000354	Isorhamnetin	*Glycyrrhiza uralensis* Fisch. (Gancao) and *Bupleurum chinense* DC. (Chaihu)
MOL002565	Medicarpin	*Glycyrrhiza uralensis* Fisch. (Gancao)

**Table 5:** The information of top 50 targets of degree values from analysis cytoscape

Name of targets	Degree	Name of targets	Degree
EGFR	85	PTGS2	47
PIK3CA	73	ALOX12	46
ADORA2A	71	JAK3	46
LCK	69	ALOX15	45
ADORA3	64	CDK4	45
PIK3R1	64	TNF	45
MMP3	63	ADAM17	44
MAPK1	62	MAPK3	44
MAPK14	60	PIK3CB	43
MMP9	60	PLA2G1B	43
SYK	60	ADORA2B	42
ALOX5	57	CYP17A1	42
F10	57	SHBG	42
HSD11B1	53	ERN1	41
MTOR	52	IL2	40
PPARG	52	MMP8	40
ABCG2	51	JAK1	39
MMP13	51	PIK3CG	39
PTGS1	51	PTPN6	38
NOS2	49	RELA	38
PARP1	49	AKR1B1	37
NR3C1	48	ITGB1	37
STAT3	48	S1PR1	37
ABCB1	47	PIK3CD	36
MAPK8	47	AKR1C3	35

**Table 6:** Molecular docking results of aim proteins and the ten ingredients of top degree

MOL ID	Molecule Name	ACE2 binding energy (kJ/moL)	S1 binding energy (kJ/moL)	3CL binding energy (kJ/moL)
MOL001689	Acacetin	-4.1	-6.14	-5.81
MOL004835	Glypallichalcone	-3.84	-6.1	-6.24
MOL000173	Wogonin	-4.1	-4.59	-6.6
MOL004905	Glyuranolide	-5.66	-6.97	-7.09
MOL004598	3',4',5',3,5,6,7-Heptamethoxy flavone	-3.03	-3.05	-4.29
MOL004828	Glepidotin A	-4.92	-4.33	-4.53
MOL004856	Gancaonin A	-4.23	-5.42	-6.44
MOL005013	18α-hydroxy glycyrrhetic acid	-5.73	-6.42	-6.85
MOL000354	Isorhamnetin	-4.5	-5.52	-6.61
MOL002565	Medicarpin	-5.39	-5.56	-5.91

## Abbreviations

JingFangBaiDu San: JFBDS
coronavirus disease 2019: COVID-19
severe acute respiratory syndrome corona virus 2: SARS-CoV-2
traditional Chinese medicine: TCM
Traditional Chinese Medicine Systems Pharmacology Database and Analysis Platform: TCMSP
severe acute respiratory syndrome coronavirus: SARS-CoV
Qing Fei Pai Du Decoction: QFPD
Lian Hua Qing Wen Capsule: LHQW
Shen fu Decoction: SF
Xuebijing injection: XBJ
Therapeutic Target Database: TTD
oral bioavailability: OB
drug-likeness: DL
protein-protein interaction: PPI
Gene Ontology: GO
Kyoto Encyclopedia of Genes and Genomes: KEGG
angiotensin converting enzyme II: ACE2
