# Supplementary material for: Potential mechanism of action of Jing Fang Bai Du San in the treatment of COVID-19 using docking and network pharmacology
Source: Int J Med Sci. 2022 Jan 1;19(2):213–24. doi: 10.7150/ijms.67116 (PMC8795796; doi:10.7150/ijms.67116)
Supplement: Supplementary file 1 — Supplementary information. [file ijmsv19p0213s1.pdf]

Ingredients of JFBDS

Bupleurum chinense DC. (Chaihu)

Mol ID	Molecule Name	OB (%)	DL
MOL001645	Linoleyl acetate	42.1	0.2
MOL002776	Baicalin	40.12	0.75
MOL000449	Stigmasterol	43.83	0.76
MOL000354	isorhamnetin	49.6	0.31
MOL000422	kaempferol	41.88	0.24
MOL004598	3,5,6,7-tetramethoxy-2-(3,4,5-trimethoxyphenyl)chromone	31.97	0.59
MOL004609	Areapillin	48.96	0.41
MOL013187	Cubebin	57.13	0.64
MOL004624	Longikaurin A	47.72	0.53
MOL004628	Octalupine	47.82	0.28
MOL004644	Sainfuran	79.91	0.23
MOL004648	Troxerutin	31.6	0.28
MOL004653	(+)-Anomalin	46.06	0.66
MOL004702	saikosaponin c_qt	30.5	0.63
MOL004718	α-spinasterol	42.98	0.76
MOL000490	petunidin	30.05	0.31
MOL000098	quercetin	46.43	0.28

Ligusticum chuanxiong Hort. (Chuanxiong)

Mol ID	Molecule Name	OB (%)	DL
MOL001494	Mandenol	42	0.19
MOL002135	Myricanone	40.6	0.51
MOL002140	Perlolyrine	65.95	0.27
MOL002151	senkyunone	47.66	0.24
MOL002157	wallichilide	42.31	0.71
MOL000359	sitosterol	36.91	0.75
MOL000433	FA	68.96	0.71

Angelica pubescens Maxim. f. *biserrata* Shan et Yuan (Duhuo)

Mol ID	Molecule Name	OB (%)	DL
MOL001941	Ammidin	34.55	0.22
MOL001942	isoimperatorin	45.46	0.23
MOL000358	beta-sitosterol	36.91	0.75
MOL003608	O-Acetylcolumbianetin	60.04	0.26
MOL004777	Angelol D	34.85	0.34
MOL004778	[(1R,2R)-2,3-dihydroxy-1-(7-methoxy-2-oxochromen-6-yl)-3-methylbutyl] (Z)-2-methylbut-2-enoate	46.03	0.34
MOL004780	Angelicone	30.99	0.19
MOL004782	[(1R,2R)-2,3-dihydroxy-1-(7-methoxy-2-oxochromen-6-yl)-3-methylbutyl] 3-methylbutanoate	45.19	0.34
MOL004792	nodakenin	57.12	0.69

Saposhnikovia divaricata (Turcz.) Schischk. (Fangfeng)

Mol ID	Molecule Name	OB (%)	DL
MOL000011	(2R,3R)-3-(4-hydroxy-3-methoxy-phenyl)-5-methoxy-2-methylol-2,3-dihydropyrano[5,6-h][1,4]benzodioxin-9-one	68.83	0.66
MOL011730	11-hydroxy-sec-o-beta-d-glucosylhamaudol_qt	50.24	0.27
MOL011732	anomalin	59.65	0.66
MOL011737	divaricatacid	87	0.32
MOL011740	divaricatol	31.65	0.38
MOL001941	Ammidin	34.55	0.22
MOL011747	ledebouriellol	32.05	0.51
MOL011749	phelloptorin	43.39	0.28
MOL011753	5-O-Methylvisamminol	37.99	0.25
MOL002644	Phellopterin	40.19	0.28
MOL000359	sitosterol	36.91	0.75
MOL000173	wogonin	30.68	0.23
MOL000358	beta-sitosterol	36.91	0.75
MOL001494	Mandenol	42	0.19
MOL001942	isoimperatorin	45.46	0.23
MOL003588	Prangenidin	36.31	0.22
MOL007514	methyl icosa-11,14-dienoate	39.67	0.23
MOL013077	Decursin	39.27	0.38

Poria cocos (Schw.) Wolf (Fuling)

Mol ID	Molecule Name	OB (%)	DL
MOL000273	(2R)-2-[(3S,5R,10S,13R,14R,16R,17R)-3,16-dihydroxy-4,4,10,13,14-pentamethyl-2,3,5,6,12,15,16,17-octahydro-1H-cyclopenta[a]phenanthren-17-yl]-6-methylhept-5-enoic acid	30.93	0.81
MOL000275	trametenolic acid	38.71	0.8
MOL000276	7,9(11)-dehydropachymic acid	35.11	0.81
MOL000279	Cerevisterol	37.96	0.77
MOL000280	(2R)-2-[(3S,5R,10S,13R,14R,16R,17R)-3,16-dihydroxy-4,4,10,13,14-pentamethyl-2,3,5,6,12,15,16,17-octahydro-1H-cyclopenta[a]phenanthren-17-yl]-5-isopropyl-hex-5-enoic acid	31.07	0.82
MOL000282	ergosta-7,22E-dien-3beta-ol	43.51	0.72
MOL000283	Ergosterol peroxide	40.36	0.81
MOL000285	(2R)-2-[(5R,10S,13R,14R,16R,17R)-16-hydroxy-3-keto-4,4,10,13,14-pentamethyl-1,2,5,6,12,15,16,17-octahydrocyclopenta[a]phenanthren-17-yl]-5-isopropyl-hex-5-enoic acid	38.26	0.82
MOL000287	3beta-Hydroxy-24-methylene-8-lanostene-21-oic acid	38.7	0.81
MOL000289	pachymic acid	33.63	0.81
MOL000290	Poricoic acid A	30.61	0.76
MOL000291	Poricoic acid B	30.52	0.75
MOL000292	poricoic acid C	38.15	0.75
MOL000296	hederagenin	36.91	0.75

MOL000300	dehydroeburicoic acid	44.17	0.83

Glycyrrhiza uralensis Fisch. (Gancao)

Mol ID	Molecule Name	OB (%)	DL
MOL001484	Inermine	75.18	0.54
MOL001792	DFV	32.76	0.18
MOL000211	Mairin	55.38	0.78
MOL002311	Glycyrol	90.78	0.67
MOL000239	Jaranol	50.83	0.29
MOL002565	Medicarpin	49.22	0.34
MOL000354	isorhamnetin	49.6	0.31
MOL000359	sitosterol	36.91	0.75
MOL003656	Lupiwighteone	51.64	0.37
MOL003896	7-Methoxy-2-methyl isoflavone	42.56	0.2
MOL000392	formononetin	69.67	0.21
MOL000417	Calycosin	47.75	0.24
MOL000422	kaempferol	41.88	0.24
MOL004328	naringenin	59.29	0.21
MOL004805	(2S)-2-[4-hydroxy-3-(3-methylbut-2-enyl)phenyl]-8,8-dimethyl-2,3-dihydropyrano[2,3-f]chromen-4-one	31.79	0.72
MOL004806	euchrenone	30.29	0.57
MOL004808	glyasperin B	65.22	0.44
MOL004810	glyasperin F	75.84	0.54
MOL004811	Glyasperin C	45.56	0.4
MOL004814	Isotrifoliol	31.94	0.42
MOL004815	(E)-1-(2,4-dihydroxyphenyl)-3-(2,2-dimethylchromen-6-yl)prop-2-en-1-one	39.62	0.35
MOL004820	kanzonols W	50.48	0.52
MOL004824	(2S)-6-(2,4-dihydroxyphenyl)-2-(2-hydroxypropan-2-yl)-4-methoxy-2,3-dihydrofuro[3,2-g]chromen-7-one	60.25	0.63
MOL004827	Semilicoisoflavone B	48.78	0.55
MOL004828	Glepidotin A	44.72	0.35
MOL004829	Glepidotin B	64.46	0.34
MOL004833	Phaseolinisoflavan	32.01	0.45
MOL004835	Glypallichalcone	61.6	0.19
MOL004838	8-(6-hydroxy-2-benzofuranyl)-2,2-dimethyl-5-chromenol	58.44	0.38
MOL004841	Licochalcone B	76.76	0.19
MOL004848	licochalcone G	49.25	0.32
MOL004849	3-(2,4-dihydroxyphenyl)-8-(1,1-dimethylprop-2-enyl)-7-hydroxy-5-methoxy-coumarin	59.62	0.43
MOL004855	Licoricone	63.58	0.47
MOL004856	Gancaonin A	51.08	0.4
MOL004857	Gancaonin B	48.79	0.45
MOL004860	licorice glycoside E	32.89	0.27
MOL004863	3-(3,4-dihydroxyphenyl)-5,7-dihydroxy-8-(3-methylbut-2-enyl)chromone	66.37	0.41

MOL004864	5,7-dihydroxy-3-(4-methoxyphenyl)-8-(3-methylbut-2-enyl)chromone	30.49	0.41
MOL004866	2-(3,4-dihydroxyphenyl)-5,7-dihydroxy-6-(3-methylbut-2-enyl)chromone	44.15	0.41
MOL004879	Glycyrin	52.61	0.47
MOL004882	Licocoumarone	33.21	0.36
MOL004883	Licoisoflavone	41.61	0.42
MOL004884	Licoisoflavone B	38.93	0.55
MOL004885	licoisoflavanone	52.47	0.54
MOL004891	shinpterocarpin	80.3	0.73
MOL004898	(E)-3-[3,4-dihydroxy-5-(3-methylbut-2-enyl)phenyl]-1-(2,4-dihydroxyphenyl)prop-2-en-1-one	46.27	0.31
MOL004903	liquiritin	65.69	0.74
MOL004904	licopyranocoumarin	80.36	0.65
MOL004905	3,22-Dihydroxy-11-oxo-delta(12)-oleanene-27-alpha-methoxycarbonyl-29-oic acid	34.32	0.55
MOL004907	Glyzaglabrin	61.07	0.35
MOL004908	Glabridin	53.25	0.47
MOL004910	Glabranin	52.9	0.31
MOL004911	Glabrene	46.27	0.44
MOL004912	Glabrone	52.51	0.5
MOL004913	1,3-dihydroxy-9-methoxy-6-benzofurano[3,2-c]chromenone	48.14	0.43
MOL004914	1,3-dihydroxy-8,9-dimethoxy-6-benzofurano[3,2-c]chromenone	62.9	0.53
MOL004915	Eurycarpin A	43.28	0.37
MOL004917	glycyroside	37.25	0.79
MOL004924	(-)-Medicocarpin	40.99	0.95
MOL004935	Sigmoidin-B	34.88	0.41
MOL004941	(2R)-7-hydroxy-2-(4-hydroxyphenyl)chroman-4-one	71.12	0.18
MOL004945	(2S)-7-hydroxy-2-(4-hydroxyphenyl)-8-(3-methylbut-2-enyl)chroman-4-one	36.57	0.32
MOL004948	Isoglycyrol	44.7	0.84
MOL004949	Isolicoflavonol	45.17	0.42
MOL004957	HMO	38.37	0.21
MOL004959	1-Methoxyphaseollidin	69.98	0.64
MOL004961	Quercetin der.	46.45	0.33
MOL004966	3'-Hydroxy-4'-O-Methylglabridin	43.71	0.57
MOL000497	licochalcone a	40.79	0.29
MOL004974	3'-Methoxyglabridin	46.16	0.57
MOL004978	2-[(3R)-8,8-dimethyl-3,4-dihydro-2H-pyrano[6,5-f]chromen-3-yl]-5-methoxyphenol	36.21	0.52
MOL004980	Inflacoumarin A	39.71	0.33
MOL004985	icos-5-enoic acid	30.7	0.2
MOL004988	Kanzonol F	32.47	0.89
MOL004989	6-prenylated eriodictyol	39.22	0.41
MOL004990	7,2',4'-trihydroxy－5-methoxy-3－arylcoumarin	83.71	0.27
MOL004991	7-Acetoxy-2-methylisoflavone	38.92	0.26

MOL004993	8-prenylated eriodictyol	53.79	0.4
MOL004996	gadelaidic acid	30.7	0.2
MOL000500	Vestitol	74.66	0.21
MOL005000	Gancaonin G	60.44	0.39
MOL005001	Gancaonin H	50.1	0.78
MOL005003	Licoagrocarpin	58.81	0.58
MOL005007	Glyasperins M	72.67	0.59
MOL005008	Glycyrrhiza flavonol A	41.28	0.6
MOL005012	Licoagroisoflavone	57.28	0.49
MOL005013	18α-hydroxyglycyrrhetic acid	41.16	0.71
MOL005016	Odoratin	49.95	0.3
MOL005017	Phaseol	78.77	0.58
MOL005018	Xambioona	54.85	0.87
MOL005020	dehydroglyasperins C	53.82	0.37
MOL000098	quercetin	46.43	0.28

Schizonepeta tenuifolia Briq. (Jingjie)

Mol ID	Molecule Name	OB (%)	DL
MOL011849	Schizonepetoside B	31.02	0.28
MOL011856	Schkuhrin I	54.45	0.52
MOL002881	Diosmetin	31.14	0.27
MOL000359	sitosterol	36.91	0.75
MOL005100	5,7-dihydroxy-2-(3-hydroxy-4-methoxyphenyl)chroman-4-one	47.74	0.27
MOL000006	luteolin	36.16	0.25
MOL000098	quercetin	46.43	0.28
MOL000358	beta-sitosterol	36.91	0.75
MOL000449	Stigmasterol	43.83	0.76
MOL001506	Supraene	33.55	0.42
MOL005043	campest-5-en-3beta-ol	37.58	0.71

Platycodon grandiflorum (Jacq.) A. DC. (Jiegeng)

Mol ID	Molecule Name	OB (%)	DL
MOL001689	acacetin	34.97	0.24
MOL004355	Spinasterol	42.98	0.76
MOL004580	cis-Dihydroquercetin	66.44	0.27
MOL005996	2-O-methyl-3—O-β-D-glucopyranosyl platycogenate A	45.15	0.25
MOL000006	luteolin	36.16	0.25
MOL006026	dimethyl 2-O-methyl-3-O-a-D-glucopyranosyl platycogenate A	39.21	0.25
MOL006070	robinin	39.84	0.71

Peucedanum praeruptorum Dunn (Qianhu),

Mol ID	Molecule Name	OB (%)	DL
MOL013076	(8S,9R)-9-hydroxy-8-(2-hydroxypropan-2-yl)-8,9-dihydrofuro[2,3-h]chromen-2-one	37.3	0.2
MOL013077	Decursin	39.27	0.38
MOL013078	praeruptorin E	51.22	0.66

MOL013079	dl-praeruptorin a	46.46	0.53
MOL013081	Decussine	39.83	0.65
MOL013083	Skimmin (8CI)	38.35	0.32
MOL013087	Peucedanocoumarin II	63.48	0.53
MOL013093	rubricauloside	58.36	0.71
MOL013094	8-[(2R)-2,3-dihydroxy-3-methyl-butyl]-5,7-dimethoxy-coumarin	48.57	0.21
MOL013095	Sporidesmin	58.31	0.76
MOL013098	[(9R)-8,8-dimethyl-2-oxo-9,10-dihydropyrano[6,5-h]chromen-9-yl] (Z)-2-methylbut-2-enoate	87.48	0.37
MOL013100	(2S)-2-(1-hydroxy-1-methyl-ethyl)-9-[(2S,3R,4R,5S,6R)-3,4,5-trihydroxy-6-methylol-tetrahydropyran-2-yl]oxy-2,3-dihydrofuro[3,2-g]chromen-7-one	45.33	0.73
MOL013101	rutarin_qt	70.1	0.2
MOL013103	532-16-1	46.57	0.44
MOL001941	Ammidin	34.55	0.22
MOL001942	isoimperatorin	45.46	0.23
MOL002644	Phellopterin	40.19	0.28
MOL000358	beta-sitosterol	36.91	0.75
MOL000359	sitosterol	36.91	0.75
MOL004653	(+)-Anomalin	46.06	0.66
MOL004792	nodakenin	57.12	0.69
MOL005100	5,7-dihydroxy-2-(3-hydroxy-4-methoxyphenyl)chroman-4-one	47.74	0.27
MOL007154	tanshinone iia	49.89	0.4
MOL000098	quercetin	46.43	0.28

Notopterygium inchum Ting ex H.T. Chang (Qianghuo)

Mol ID	Molecule Name	OB (%)	DL
MOL001941	Ammidin	34.55	0.22
MOL011962	6'-Feruloylnodakenin	32.02	0.67
MOL011963	8-geranoxy-5-methoxypsoralen	40.97	0.5
MOL011968	coumarin,glycoside	33.07	0.78
MOL011969	Demethylfuropinnarin	41.31	0.21
MOL011971	diversoside_qt	67.57	0.31
MOL011975	notoptol	62.97	0.48
MOL001951	Bergaptin	41.73	0.42
MOL001956	Cnidilin	32.69	0.28
MOL000359	sitosterol	36.91	0.75
MOL004792	nodakenin	57.12	0.69
MOL000358	beta-sitosterol	36.91	0.75
MOL001942	isoimperatorin	45.46	0.23
MOL002644	Phellopterin	40.19	0.28
MOL002881	Diosmetin	31.14	0.27

Citrus aurantium L. (Zhiqiao)

Mol ID	Molecule Name	OB (%)	DL

MOL013381	Marmin	38.23	0.31
MOL002341	Hesperetin	70.31	0.27
MOL000358	beta-sitosterol	36.91	0.75
MOL004328	naringenin	59.29	0.21
MOL005828	nobiletin	61.67	0.52

Intersective targets

target	gene name
P15121	AKR1B1
P00533	EGFR
P09917	ALOX5
P05164	MPO
P27986	PIK3R1
P29274	ADORA2A
P45452	MMP13
P08254	MMP3
P16050	ALOX15
P33527	ABCC1
P14780	MMP9
P18054	ALOX12
P08183	ABCB1
P04054	PLA2G1B
Q16678	CYP1B1
Q9UNQ0	ABCG2
P52895	AKR1C2
Q04828	AKR1C1
P42330	AKR1C3
P17516	AKR1C4
P43405	SYK
Q15746	MYLK
P48736	PIK3CG
P35869	AHR
P09874	PARP1
P02766	TTR
P39900	MMP12
O14746	TERT
Q07820	MCL1
P00747	PLG
P35354	PTGS2
P13569	CFTR
P05093	CYP17A1
P04278	SHBG
P33261	CYP2C19
P08185	SERPINA6
P11413	G6PD

P11473	VDR
P28845	HSD11B1
P29350	PTPN6
P04150	NR3C1
P35228	NOS2
P80365	HSD11B2
P37231	PPARG
P10415	BCL2
P25116	F2R
P49682	CXCR3
P21730	C5AR1
P06239	LCK
Q16539	MAPK14
P43235	CTSK
Q9HC29	NOD2
P25105	PTAFR
P43490	NAMPT
P00374	DHFR
P01375	TNF
P60568	IL2
P0DMS8	ADORA3
P31213	SRD5A2
P27487	DPP4
P07550	ADRB2
Q96EB6	SIRT1
P13497	BMP1
P13726	F3
P08246	ELANE
P07333	CSF1R
P07711	CTSL
P62937	PPIA
P52333	JAK3
P00742	F10
P55072	VCP
P25025	CXCR2
P42574	CASP3
P06756	ITGAV
Q13946	PDE7A
P30281	CCND3
O75762	TRPA1
O14920	IKBKB
Q16665	HIF1A
P32246	CCR1
O75469	NR1I2
P40189	IL6ST
P54707	ATP12A

Q01469	FABP5
P27361	MAPK3
P00491	PNP
Q14790	CASP8
P29466	CASP1
P42345	MTOR
P22894	MMP8
P23458	JAK1
Q9NZJ5	EIF2AK3
P78536	ADAM17
Q92793	CREBBP
Q16853	AOC3
O00329	PIK3CD
P42338	PIK3CB
P11802	CDK4
P04818	TYMS
P23219	PTGS1
P00749	PLAU
Q96P20	NLRP3
Q06187	BTK
P40763	STAT3
P15144	ANPEP
P42226	STAT6
P49137	MAPKAPK2
P29275	ADORA2B
P27815	PDE4A
Q02763	TEK
P45983	MAPK8
P41597	CCR2
P51677	CCR3
Q15722	LTB4R
P07858	CTSB
P12821	ACE
P21453	S1PR1
P42892	ECE1
P05177	CYP1A2
P42336	PIK3CA
P51686	CCR9
O60760	HPGDS
O15111	CHUK
P29474	NOS3
P28482	MAPK1
P17252	PRKCA
P04406	GAPDH
Q92934	BAD
P05362	ICAM1

P19320	VCAM1
Q05655	PRKCD
O00443	PIK3C2A
P05412	JUN
P31751	AKT2
P04798	CYP1A1
O75460	ERN1
O95470	SGPL1
P55212	CASP6
Q9Y5Y4	PTGDR2
P05556	ITGB1
Q99808	SLC29A1
P20701	ITGAL
P16591	FER
P08922	ROS1
P05771	PRKCB
Q9UHD2	TBK1
Q9NWZ3	IRAK4
P19525	EIF2AK2
P05107	ITGB2
P13612	ITGA4
P60033	CD81
Q4U2R8	SLC22A6
P25106	ACKR3
P11712	CYP2C9
P08684	CYP3A4
P47712	PLA2G4A
P03951	F11
Q9NR96	TLR9
P14174	MIF
Q02127	DHODH
P00750	PLAT
P15311	EZR
P09211	GSTP1
P49763	PGF
P15692	VEGFA
P05121	SERPINE1
Q07817	BCL2L1
P19838	NFKB1
Q04206	RELA
P12644	BMP4
P06734	FCER2
P61073	CXCR4
P42785	PRCP
Q6ZVD8	PHLPP2
P00403	MT-CO2

P08311	CTSG
P04792	HSPB1
P01137	TGFB1
Q02156	PRKCE
P20648	ATP4A
P42224	STAT1
P10635	CYP2D6
P09038	FGF2
P11021	HSPA5
P00813	ADA
Q9NZQ7	CD274
O00182	LGALS9
P55085	F2RL1
Q9BZP6	CHIA
O00206	TLR4
P19438	TNFRSF1A
Q13526	PIN1

Targets of COVID-19

Gene name	ID
CD19	P15391
CRP	P02741
IFNG	P01579
IL6	P05231
TWIST1	Q15672
CARTPT	Q16568
IL1B	P01584
CXCL1	P09341
CCL2	P13500
CCL11	P51671
IL18	Q14116
CCL17	Q92583
ITGB2	P05107
TLR4	O00206
IL17A	Q16552
TNF	P01375
IL13	P35225
CSF2	P04141
IL4	P05112
CXCR2	P25025
IL33	O95760
CXCL5	P42830
HMOX1	P09601
MYD88	Q99836
TGFB1	P01137
CCL24	O00175

CASP1	P29466
SIRT1	Q96EB6
IL6ST	P40189
CD40LG	P29965
AHR	P35869
CCL20	P78556
CXCR3	P49682
PARP1	P09874
POMC	P01189
CX3CL1	P78423
IL1R1	P14778
IL1R2	P27930
CCL4	P13236
ABCF1	Q8NE71
IL5RA	Q01344
CCL19	Q99731
CCR1	P32246
CCR2	P41597
CCL7	P80098
CCR6	P51684
CCL8	P80075
CXCL9	Q07325
IL2RB	P14784
AGT	P01019
SPP1	P10451
PF4	P02776
CCR9	P51686
MYLK	Q15746
IL1RN	P18510
ICAM1	P05362
CXCL2	P19875
IL1A	P01583
IKBKB	O14920
MUC2	Q02817
ADRB2	P07550
REG3A	Q06141
ITGA4	P13612
C3	P01024
CCL22	O00626
SLC11A2	P49281
NFKB1	P19838
SFTPC	P11686
SFTPA2	Q8IWL1
FER	P16591
ICOS	Q9Y6W8
ADA	P00813

JAK3	P52333
CR2	P20023
CARD11	Q9BXL7
ZAP70	P43403
CHD7	Q9P2D1
SLC35C1	Q96A29
RNF125	Q96EQ8
DCLRE1C	Q96SD1
SELENON	Q9NZV5
TNFRSF13B	O14836
RAG2	P55895
PNP	P00491
NFKB2	Q00653
ZBTB24	O43167
MBL2	P11226
TNFSF12	O43508
LIG4	P49917
SLC35A1	P78382
IL10	P22301
CXCL8	P10145
IL7R	P16871
PRKCD	Q05655
IL2RG	P31785
OFD1	O75665
NIPBL	Q6KC79
DNAI1	Q9UI46
IL21R	Q9HBE5
RAG1	P15918
SERPINE1	P05121
SGCG	Q13326
CASP8	Q14790
DNMT3B	Q9UBC3
LRBA	P50851
MS4A1	P11836
TERT	O14746
DOCK8	Q8NF50
CFB	P00751
BTK	Q06187
TNFRSF13C	Q96RJ3
ACTA1	P68133
ACP5	P13686
CD81	P60033
ACE	P12821
STAT3	P40763
CFTR	P13569
SLC9A6	Q92581

MMP9	P14780
DHFR	P00374
MIP	P30301
TLR2	O60603
PTGS2	P35354
DHPS	P49366
IL5	P05113
SFTPB	P07988
HIF1A	Q16665
SLC27A5	Q9Y2P5
CD44	P16070
PTGDS	P41222
SFTPD	P35247
CEL	P19835
DECR1	Q16698
MUC5AC	P98088
NFE2L2	Q16236
VEGFA	P15692
SCGB1A1	P11684
SARS1	P49591
MT-CO2	P00403
PARP9	Q8IXQ6
ELANE	P08246
FCGR2A	P12318
CAV1	Q03135
FOXM1	Q08050
PDE4A	P27815
NLRP3	Q96P20
DEFB4A	O15263
PIK3CG	P48736
HPGDS	O60760
ADAM17	P78536
JUN	P05412
PRCP	P42785
BMP1	P13497
SOCS3	O14543
ATP12A	P54707
PPARG	P37231
TIMP1	P01033
ALDH7A1	P49419
AOC3	Q16853
LPAR2	Q9HBW0
NFKBIA	P25963
APOE	P02649
CLOCK	O15516
MMP8	P22894

NR1I2	O75469
OSM	P13725
NAMPT	P43490
MMP12	P39900
ALOX5	P09917
MPO	P05164
LCT	P09848
LCN2	P80188
JUND	P17535
PLAU	P00749
JUNB	P17275
ADORA2B	P29275
APOA1	P02647
TLR3	O15455
F2R	P25116
PRMT1	Q99873
CD274	Q9NZQ7
HLA-DRB1	P01911
CXCL12	P48061
CREBBP	Q92793
IL22	Q9GZX6
F2RL1	P55085
GZMB	P10144
ROS1	P08922
IL23A	Q9NPF7
IL17D	Q8TAD2
TREM1	Q9NP99
SMOX	Q9NWM0
FOS	P01100
PGPEP1	Q9NXJ5
MYDGF	Q969H8
FCGR1A	P12314
GABPA	Q06546
CD86	P42081
IL2	P60568
NKX2-1	P43699
CD40	P25942
TP53	P04637
RBM45	Q8IUH3
FOSB	P53539
ID2	Q02363
IRAK3	Q9Y616
CD14	P08571
CYBB	P04839
VIP	P01282
TRPA1	O75762

VIM	P08670
SEMA7A	O75326
VDR	P11473
VASP	P50552
PIAS1	O75925
TIRAP	P58753
VCP	P55072
VCAM1	P19320
TLR5	O60602
TIMELESS	Q9UNS1
ARHGEF5	Q12774
TNFSF10	P50591
TNFRSF6B	O95407
AIMP2	Q13155
FOSL1	P15407
NRP1	O14786
TNFSF4	P23510
CXCR4	P61073
NR0B2	Q15466
TNFRSF25	Q93038
TTF1	Q15361
TXN	P10599
TYMS	P04818
ARHGEF7	Q14155
SGPL1	O95470
TRAF1	Q13077
TNFRSF11A	Q9Y6Q6
GPRC5A	Q8NFJ5
ACKR3	P25106
PINK1	Q9BXM7
SLC52A2	Q9HAB3
ZC3H12A	Q5D1E8
SESN2	P58004
JAM3	Q9BX67
ITCH	Q96J02
RPAIN	Q86UA6
IL25	Q9H293
CLEC7A	Q9BXN2
CEMIP	Q8WUJ3
EPG5	Q9HCE0
NLRC4	Q9NPP4
ACE2	Q9BYF1
CRLF2	Q9HC73
NOD2	Q9HC29
SEMA4A	Q9H3S1
RETNLB	Q9BQ08

TSLP	Q969D9
ESAM	Q96AP7
IL27	Q8NEV9
LCLAT1	Q6UWP7
MYO18A	Q92614
SFTPA1	Q8IWL2
FBXO15	Q8NCQ5
TICAM1	Q8IUC6
NLRP12	P59046
CLEC6A	Q6EIG7
BUD23	O43709
UCN3	Q969E3
SCGB3A2	Q96PL1
TRIM69	Q86WT6
PLEKHA7	Q6IQ23
SLC52A1	Q9NWF4
FCGR2C	P31995
CCL26	Q9Y258
KLF2	Q9Y5W3
UBAC1	Q9BSL1
FST	P19883
ATG7	O95352
PROCR	Q9UNN8
CXCL13	O43927
AHSA1	O95433
TLR6	Q9Y2C9
PLXNC1	O60486
SCAF11	Q99590
ARHGEF2	Q92974
XPR1	Q9UBH6
KLF4	O43474
RAB9A	P51151
GRAP2	O75791
GDF15	Q99988
LRPPRC	P42704
MASP2	O00187
MORF4L1	Q9UBU8
PTGDR2	Q9Y5Y4
FOXP3	Q9BZS1
ASCC1	Q8N9N2
IRAK4	Q9NWZ3
HSPA14	Q0VDF9
NELFCD	Q8IXH7
WNT4	P56705
CRLS1	Q9UJA2
BLNK	Q8WV28

CCDC22	O60826
PHLPP2	Q6ZVD8
ICOSLG	O75144
TLR9	Q9NR96
LY96	Q9Y6Y9
RNF19A	Q9NV58
POLDIP2	Q9Y2S7
HAVCR1	Q96D42
CHIA	Q9BZP6
FEV	Q99581
CYP2E1	P05181
MS4A2	Q01362
FCGR2B	P31994
FCGR3A	P08637
FCGR3B	O75015
FGF7	P21781
FGG	P02679
FOXF1	Q12946
FOXO3	O43524
FABP5	Q01469
F11	P03951
F3	P13726
DUSP1	P28562
ECE1	P42892
S1PR1	P21453
LPAR1	Q92633
ELN	P15502
EMP1	P54849
SLC29A1	Q99808
ETS2	P15036
FXN	Q16595
MTOR	P42345
CFHR1	Q03591
HGF	P14210
HLA-DPB1	P04440
HMGB1	P09429
SLC29A2	Q14542
HP	P00738
HSPA1A	P0DMV8
HSPA1B	P0DMV9
CFH	P08603
HCCS	P53701
GZMA	P12544
ACKR1	Q16570
GATA3	P23771
GCLC	P48506

GCLM	P48507
NR3C1	P04150
GSTM1	P09488
GSTP1	P09211
GSTT1	P30711
HSPA4	P34932
CYP1A1	P04798
ABCA3	Q99758
APC	P25054
BIRC3	Q13489
APRT	P07741
ATF3	P18847
ATP4A	P20648
BAX	Q07812
BCL2	P10415
BDNF	P23560
AOC2	O75106
ANXA5	P08758
ANGPT1	Q15389
ADAM8	P78325
ADORA2A	P29274
ADORA3	P0DMS8
AGER	Q15109
AKT2	P31751
AKR1B1	P15121
ALOX12	P18054
ALOX15	P16050
BMP4	P12644
BRCA1	P38398
CCR8	P51685
LTB4R	Q15722
COX8A	P10176
CRK	P46108
MAPK14	Q16539
CSF3	P09919
CTSG	P08311
CTSK	P43235
CCR3	P51677
CHUK	O15111
CDC42	P60953
BRCA2	P51587
C1QBP	Q07021
C3AR1	Q16581
C5AR1	P21730
CD2	P06729
TNFRSF8	P28908

ENTPD1	P49961
CD68	P34810
CYLD	Q9NQC7
CENPC	Q03188
MAPK3	P27361
MAPK8	P45983
PRSS2	P07478
PTAFR	P25105
PTGS1	P23219
PTPN6	P29350
MOK	Q9UQ07
RAP1B	P61224
MAPK1	P28482
PLG	P00747
PGF	P49763
PIK3CA	P42336
PIK3CB	P42338
PIK3CD	O00329
PIN1	Q13526
PLA2G1B	P04054
PLA2G4A	P47712
PLAT	P00750
RNASE3	P12724
RPS19	P39019
SELPLG	Q14242
ISG20	Q96AZ6
SMPD2	O60906
SYK	P43405
TAGLN	Q01995
TEK	Q02763
TFPI	P10646
SELP	P16109
SELL	P14151
CCL18	P55774
S100A1	P23297
S100A9	P06702
S100A12	P80511
S100B	P04271
ATXN7	O15265
CCL3	P10147
CCL5	P13501
CCL13	Q99616
TGFA	P01135
PDE7A	Q13946
HSPA5	P11021
IRF6	O14896

IRF7	Q92985
ITGA5	P08648
ITGAE	P38570
ITGAV	P06756
ITGAX	P20702
LAMC2	Q13753
LGALS9	O00182
IRF3	Q14653
CXCL10	P02778
IL16	Q14005
HSPD1	P10809
IFNA1	P01562
IGF1	P05019
IL2RA	P01589
IL7	P13232
IL9	P15248
IL15	P40933
LPA	P08519
LSP1	P33241
MT-CYB	P00156
NEO1	Q92859
NFATC2	Q13469
NINJ1	Q92982
NOS2	P35228
NOS3	P29474
NTF3	P20783
PEBP1	P30086
MT-CO1	P00395
MT1E	P04732
MRC1	P22897
NBR1	Q14596
MCAM	P43121
MCL1	Q07820
MEFV	O15553
MIF	P14174
MMP3	P08254
MMP13	P45452
PDCD1	Q15116
IL3	P08700
IL12A	P29459
IL11	P20809
IL12B	P29460
IL19	Q9UHD0
IL20	Q9NYY1
IL21	Q9HBE4
IL24	Q13007

IL26	Q9NPH9
IL28RA	H2DF04
IL31RA	Q8NI17
IL31	Q6EBC2
IL32	P24001
IL36B	Q9NZH7
IL36A	Q9UHA7
IL37	Q9NZH6
CCL1	P22362
CCL14	Q16627
CCL15	Q16663
CCL16	O15467
CCL21	O00585
CCL23	P55773
CCL25	O15444
CCL27	Q9Y4X3
CCL28	Q9NRJ3
CXCL3	P19876
CXCL6	P80162
CXCL11	O14625
CXCL14	O95715
CXCL16	I3L333
XCL1	P47992
XCL2	Q9UBD3
CSF3R	Q99062
EPO	P01588
LIF	P15018
CSF1	P09603
CSF2RB	P32927
CSF2RA	P15509
CSF1R	P07333
EPOR	P19235
LIFR	P42702
TNFRSF1B	P20333
TNFRSF1A	P19438
DHODH	Q02127
CYP2C9	P11712
CYP3A4	P08684
ORM1	P02763
ABCB1	P08183
CYP3A5	P20815
SLC22A6	Q4U2R8
ABCB11	O95342
ABCC1	P33527
CYP2C19	P33261
TRDMT1	O14717

CYP2D6	P10635
CYP4A11	Q02928
SERPINA6	P08185
SLCO1A2	P46721
ALB	P02768
CYP3A43	Q9HB55
CYP3A7	P24462
CYP2A6	P11509
CYP1B1	Q16678
CYP2B6	P20813
CYP2C8	P10632
HSD11B1	P28845
CYP1A2	P05177
KRT7	P08729
ANXA1	P04083
NR0B1	P51843
ABCC2	Q92887
ABCG2	Q9UNQ0
CYP17A1	P05093
HSD11B2	P80365
SLC22A8	Q8TCC7
SHBG	P04278
AKR1D1	P51857
SRD5A2	P31213
AKR1C1	Q04828
AKR1C2	P52895
AKR1C3	P42330
AKR1C4	P17516
SLC15A1	P46059
SLC15A2	Q16348
SLC22A11	Q9NSA0
ABCC4	O15439
TPMT	P51580
SLC22A5	O76082
SLC22A7	Q9Y694
GLUL	P15104
A2M	P01023
IDE	P14735
AMD1	P17707
CCL4L1	Q8NHW4
HLA-DRB1	P01912
HLA-B	P01889
CASP3	P42574
STAT1	P42224
DPP4	P27487
TMPRSS2	O15393

HLA-C	P10321
CLEC4M	Q9H2X3
MX1	P20591
CASP6	P55212
BAK1	Q16611
EGFR	P00533
IFNL1	Q8IU54
SPIDR	Q14159
POLD1	P28340
IFNAR1	P17181
CCR5	P51681
GPT	P24298
IFNB1	P01574
ALB	Q8TES7
FGF2	P09038
CD79A	P11912
TTR	P02766
CTSL	P07711
ANPEP	P15144
PIK3R1	P27986
CD209	Q9NNX6
DDX58	O95786
FURIN	P09958
HLA-A	P04439
IFIH1	Q9BYX4
CDK4	P11802
CAT	P04040
CAT	Q6IB77
SOD1	P00441
G6PD	P11413
CTRL	P40313
CD4	P01730
TF	P02787
PIK3C2A	O00443
RELA	Q04206
NOS2	P60321
EIF2AK2	P19525
GAPDH	P04406
TRAF6	Q9Y4K3
NOS3	P60323
CTSB	P07858
F8	P00451
SH2D3A	Q9BRG2
CREB1	P16220
TMPRSS11D	O60235
BMP6	P22004

SMAD3	P84022
IFITM3	Q01628
BAD	Q92934
CANX	P27824
EGR1	P18146
ITGB1	P05556
STING1	Q86WV6
CD34	P28906
RB1	P06400
HSPB1	P04792
EEF1A1	P68104
TOLLIP	Q9H0E2
EZR	P15311
TRAF3	Q13114
ICAM3	P32942
SMAD7	O15105
ANXA2	P07355
ITGAL	P20701
STAT6	P42226
HELLS	Q9NRZ9
CP	P00450
RPS27A	P62979
LMAN1	P49257
CEACAM5	P06731
PRKCA	P17252
EIF2S1	P05198
CLEC12A	Q5QGZ9
SUMO1	P63165
UBB	P0CG47
MAPKAPK2	P49137
CD3D	P04234
CHKB	Q9Y259
PPIA	P62937
RUNX1	Q01196
BCL2L1	Q07817
IRF1	P10914
CST3	P01034
F10	P00742
F10	Q9BQ89
TFRC	P02786
CBL	P22681
MAVS	Q7Z434
KPNB1	Q14974
SLC17A5	Q9NRA2
ARF1	P84077
GRB2	P62993

CD3E	P07766
ATF2	P15336
CEACAM3	P40198
HAVCR2	Q8TDQ0
JAK1	P23458
NPM1	P06748
PYCARD	Q9ULZ3
TBK1	Q9UHD2
VHL	P40337
KPNA2	P52292
RELB	Q01201
HFE	Q30201
FCER2	P06734
BECN1	Q14457
RAPGEF3	O95398
ISG15	P05161
PML	P29590
PRKCE	Q02156
CEACAM1	P13688
ERN1	O75460
IFITM1	P13164
NPTX1	Q15818
TLR10	Q9BXR5
SLC40A1	Q9NP59
LCK	P06239
EIF2AK3	Q9NZJ5
POU5F1	Q01860
VAPA	Q9P0L0
CARD9	Q9H257
TRIM25	Q14258
HNRNPA1	P09651
CCND3	P30281
MYOM2	P54296
PRKRA	O75569
LCN1	P31025
EIF4E	P06730
ICAM2	P13598
BST2	Q10589
IFITM2	Q01629
KPNA4	O00629
USP7	Q93009
DROSHA	Q9NRR4
CD46	P15529
AHSG	P02765
BAG3	O95817
TMPRSS11A	Q6ZMR5

APOD	P05090
PRKCB	P05771
RHOB	P62745
ITGA6	P23229
CALM1	P0DP23
STAT2	P52630
BCL2L2	Q92843
IFI27	P40305
PSMC6	P62333
TFR2	Q9UP52
SPI1	P17947
IGKC	P01834
PHB2	Q99623
CD151	P48509
ITGA1	P56199
FAH	P16930
NUDT2	P50583
AQP1	P29972
TMPRSS13	Q9BYE2
CD3G	P09693
PCSK5	Q92824
CBLB	Q13191
PIK3R2	O00459
TMEM233	B4DJY2
ANXA11	P50995
CLEC4D	Q8WXI8
NMRAL1	Q9HBL8
OAS1	P00973
SLC39A14	Q15043
OR8U9	P0C7N5
C8G	P07360
